# Preparation and Evaluation of Nanofibrous and Film-Structured Ciprofloxacin Hydrochloride Inserts for Sustained Ocular Delivery: Pharmacokinetic Study in Rabbit’s Eye

**DOI:** 10.3390/life13040913

**Published:** 2023-03-30

**Authors:** Shiva Taghe, Shahla Mirzaeei, Arian Ahmadi

**Affiliations:** 1 Pharmaceutical Sciences Research Center, Rahesh Daru Novine, Kermanshah 6715847141, Iran; 2 Student Research Committee, Kermanshah University of Medical Sciences, Kermanshah 6715847141, Iran; 3 Pharmaceutical Sciences Research Center, Health Institute, Kermanshah University of Medical Sciences, Kermanshah 6715847141, Iran; 4 Nano Drug Delivery Research Center, Health Technology Institute, Kermanshah University of Medical Sciences, Kermanshah 6715847141, Iran

**Keywords:** antibiotics, ciprofloxacin hydrochloride, electrospinning, nanofibers, ocular drug delivery, ocular inserts

## Abstract

Conventional anti-infective eye drops are the most common forms of drugs prescribed for the management of topical ocular infections. Despite their convenience, topical eye drops face multiple challenges, including limited bioavailability and repetitive administration. The present study aimed to prepare, evaluate, and compare film-structured and nanofibrous ocular inserts using biocompatible polymers of polyvinyl alcohol (PVA) and polycaprolactone (PCL) to achieve sustained ciprofloxacin Hydrochloride (CIP) delivery. The nanofibrous formulations were prepared by electrospinning and glutaraldehyde crosslinking while the film formulation was prepared by solvent casting. Nanofibrous inserts had mean diameters in the range 330–450 nm. Both film and nanofibrous inserts were strong, although the nanofibers had higher flexibility. In vitro antibacterial efficacy against *Staphylococcus aureus* and *Escherichia coli* was observed for all formulations and cell viability of more than 70% confirmed their non-toxicity. In vitro release studies showed prolonged release of 2 days for the film and 5 days for the nanofibers compared with a 10-h release of CIP from the eye drop. Pharmacokinetic studies of rabbits’ eyes showed 4.5–5-folds higher AUC for the nanofiber formulations compared with the eye drop. Thus, prolonged-release film-structured and nanofibrous inserts are suitable carriers for ocular delivery of CIP.

## 1. Introduction

The eye is an important organ as it is the sensory part of the visual system responsible for establishing the sense of sight; hence, multiple natural mechanisms such as tear film turnover and blinking are responsible for protecting the eye from harmful external agents [1,2]. Although beneficial, these defense mechanisms make ocular drug delivery challenging due to the rapid elimination of drugs from the cornea [3]. Therefore, most of the conventional formulations, including eye drops and ointments, require repetitive administration, which can reduce patient acceptance and increase treatment failure due to the high probability of missed doses [4].

Additionally, eye drop solutions are unstable and require additional preservatives to prevent microbial contamination. Formulating the drug as an aqueous-free and preservative-free insert comes with multiple advantages such as higher drug stability and lower preservative-induced toxicity [5].

Novel drug delivery systems such as ocular inserts and nano-structured preparations have been developed to overcome the common limitations of conventional ocular drug delivery systems by enhancing the drug’s contact time with the cornea and increasing intraocular bioavailability [6,7]. One of the most important benefits of prepared inserts is the convenience and ease of administration to the conjunctival sac [6,8]. Nanofibrous inserts are one of the most favorable systems for topical ocular delivery owing to their unique properties such as high surface-to-volume ratio, porous structure, strength, flexibility, and the ability for controlled release of the drug [9]. These systems are also preferred because of their potential to be fabricated from various biocompatible polymers. Polycaprolactone (PCL) [5] and polyvinyl alcohol (PVA) [10] are examples of polymers that have been widely used for the preparation of drug delivery systems owing to their unique and beneficial characteristics [11]. Polymeric films are also promising systems for modified release of the drug as they are cost-effective and have convenient fabrication process [12]. Typically, nanofibrous inserts are more favorable than films due to their higher flexibility, highly controlled release, and water-absorbing properties [13,14].

Among the fluoroquinolones, ciprofloxacin hydrochloride (CIP) has the highest efficacy against *S. aureus*, the most important microorganism; therefore, CIP is the most recommended topical fluoroquinolone antibiotic for the treatment of bacterial conjunctivitis [15]. Despite being efficacious, CIP eye drops require repetitive administration to remain at therapeutic concentrations in the eyes. This can reduce patient acceptance and increase the risk of treatment failure following an increased number of missed doses.

The present study aimed to design and develop film-structured and nanofibrous CIP inserts for topical administration in the form of a sustained-release drug delivery for the treatment of topical ocular infections. Following physicochemical characterization and safety evaluation, the prepared inserts were inserted non-invasively into the rabbit’s eye sac for in vivo evaluation of the formulations. These formulations were expected to provide higher intraocular bioavailability and concentrations in the tear fluid, sustain drug release, and provide targeted drug delivery to the eye.

## 2. Materials and Methods

### 2.1. Materials

PVA and PCL were purchased from Sigma Aldrich (Steinheim, Germany). CIP was procured from Cipla (Mumbai, India). Sodium hydroxide, orthophosphoric acid, dichloromethane (DCM), dimethylformamide (DMF), acetonitrile, triethylamine buffer, and glutaraldehyde (GA) were purchased from Merck (Darmstadt, Germany).

### 2.2. Preparation of Ocular Inserts

#### 2.2.1. Film Formulation

To prepare CIP-F film formulation, a certain volume of PVA was first dissolved in distilled water via magnetic stirring (100 rpm) at 40 °C for 24 h to obtain a 10% *w*/*v* solution under aseptic conditions. One hundred and fifty milligrams of CIP dissolved in a specific volume of acetic acid (1% *v*/*v* aqueous solution) was added to the PVA solution. CIP-F formulation was prepared using film casting method by pouring the solution into a petri dish and letting it dry at 60 °C for 10 h.

#### 2.2.2. Nanofibrous Formulations

##### CIP-O

A 10% *w*/*v* concentration of PVA solution was prepared in distilled water. CIP (150 mg) solution was prepared using the minimum possible volume of acetic acid (1% *v*/*v* aqueous solution) and added to the PVA solution under continuous stirring until a clear solution was obtained. PVA/CIP solution was loaded into an injector with a polyethylene needle (internal diameter of 0.1 mm) and then injected, at a 0.5 mL/h flow rate, into a rotary collector wrapped with an aluminum foil. A high voltage of 15 kV was applied between the injector and collector.

##### CIP-OG

The nanofiber was placed in a desiccator containing GA aqueous solution (50% *v*/*v*) and exposed to the GA vapor at 25 °C for 10 h. After crosslinking, the formulation was removed and subjected to further studies.

##### CIP-S

CIP-S formulation with a sandwich structure was prepared through three-step consecutive electrospinning of different polymeric solutions. The outer layers contain drug-free PCL fibers surrounding the middle drug-loaded PVA layer. The PCL solution was prepared by dissolving the proper amount of PCL in a solvent mixture of DCM:DMF (7:3 *v*/*v* ratio) and stirring in a closed glass vial for 6 h at room temperature. Ten milliliters of this solution was ejected, at a 0.5 mL/h flow rate, towards a collector at a high voltage of 18 kV. After deposition of the first nanofibrous layer, the CIP-containing PVA layer (with the same components as the CIP-O electrospinning solution) was loaded into the injector and injected at the same flow rate as the first layer. The process was followed by adding another round of drug-free PCL nanofibers to form the final and outermost layer.

### 2.3. High-Performance Liquid Chromatography (HPLC)

Chromatographic separation was performed using a Knauer-V7603 chromatograph (Berlin, Germany) equipped with a UV detector (Knauer Smartline 2500, Berlin, Germany) set at a maximum wavelength of 271 nm. The analysis C18 column (250 × 4.6 mm ID) supplied by Analysentechnik (Mainz, Germany) was used. A mixture of acetonitrile and triethylamine buffer solution (0.1% *v*/*v*, 30:70 *v*/*v* ratio) with pH adjusted to 3.0 with phosphoric acid was used as the mobile phase. Standard CIP solutions with different concentrations were prepared and injected into the device at a flow rate of 1.5 mL/min.

### 2.4. Physicochemical Characterization of Ocular Inserts

#### 2.4.1. Weight, Thickness, and Drug Content Uniformity

Weight uniformity testing was performed by calculating the differences in the weights of three similar pieces cut from different sections of each insert measured using a digital balance. Additionally, the thickness of the inserts were measured at different points of the mat using the Tork Craft Digital Micrometer 0–25 mm (ME30025, Quanzhou, China). Mean values were then calculated. To analyze the drug content uniformity of the CIP-F, CIP-O, and CIP-OG, a certain quantity of sample was dissolved in 2 mL of sterilized PBS containing 1% *v*/*v* acetic acid in a sealed glass flask for 30 min under magnetic stirring (100 rpm) until a transparent solution was obtained. The CIP-S formulation was first dissolved in an organic solvent containing a DCM/DMF mixture (7:3 *v*/*v* ratio) to remove the PCL coating layer, leaving only the PVA solid layer containing the drug, which was collected through centrifugation at 3000 rpm for 15 min and removed from the DCM/DMF solvent containing PCL. The layer containing the drug was then placed in an aqueous solvent (PBS) containing 1% *v*/*v* acetic acid to measure the CIP loaded in the PVA layer. Finally, CIP values for three samples of each formula were measured using HPLC, and drug content was calculated.

#### 2.4.2. Folding Endurance and Tensile Strength Testing

Folding endurance shows the flexibility of nanofibers and films and indicates their ability to resist tearing while stretching. Insert pieces with similar dimensions (2 × 3 cm^2^) were cut and placed between the thumb and fingers and folded in a 180° angle. This process was continued until the insert tore or cracked. The mean number of successful folding from three replicates was calculated and reported. To investigate the strength of inserts, the tensile strength test was performed. Initially, 3.0 × 1.5 cm^2^ pieces with a thickness of approximately 0.2 mm were cut from different insert formulations. Both ends of the sample were clamped between a pair of gripes of a laboratory-assembled device. The sample was pulled at a constant rate of 5 mm/min until it tore. The maximum resisted stress was reported as the tensile strength and elongation was investigated as an indicator of flexibility.

#### 2.4.3. Swelling, Moisture Loss, and Uptake Tests

To examine the swelling of inserts when exposed to aqueous media, pre-weighed inserts were immersed in a plate containing phosphate buffer (PBS, pH 7.4) at 37 °C. The inserts were removed from the PBS medium at regular intervals and re-weighed after eliminating excess surface medium using a filter paper. The degree of swelling was calculated according to Equation (1).

Swelling Percentage = (Final weight − Initial weight)/(Initial weight) × 100(1)

To determine the stability of the inserts under dry and humid conditions, a specific amount of each insert was placed in a desiccator containing anhydrous calcium chloride and aluminum chloride with a moisture content of 79.5%. After three days (72 h), the inserts were removed from the desiccator and weighed again, and the percentage of moisture loss was estimated using Equation (2).

Moisture loss or uptake (%) = (Initial weight − Final weight)/(Initial weight) × 100(2)

### 2.5. Antibacterial Efficacy

Initially, lyophilized Gram-positive *S. aureus* (ATCC 6538) and Gram-negative *E. coli* (ATCC 25922) bacteria were incubated separately in 9 mL of TSB at 36 °C for 24 h. Bacterial colonies were then diluted 1000 times in PBS. The prepared bacterial suspension was uniformly spread on TSA plates and similar pieces of inserts containing 1000 µg of the drug were placed onto the inoculated TSA plates and then incubated at 36 °C for another 24 h. A caliper was used to measure the diameters of the inhibited zones of growth surrounding the inserts and the average measurement was taken.

### 2.6. Fourier-Transform Infrared (FTIR) Spectroscopy

FTIR spectra were obtained for samples using the Shimadzu IR PRESTIGE-21 spectrophotometer (Tokyo, Japan). To prepare KBr pellets, the samples were ground with KBr powder and then manually compressed under 9 tons of pressure.

### 2.7. Morphology Characterization

Scanning electron microscopy (SEM) was performed to investigate the morphological characteristics of the prepared film and nanofibrous inserts. The surface properties of samples were imaged after placing a certain volume of gold-coated sample in the chamber of the SEM device (FEI/Quanta 450 FEG). The accelerating voltage in the vacuum chamber was set at 15–30 kV. The diameters of fibers were measured using ImageJ software and the mean of 20 strands was reported.

### 2.8. In Vitro Release Study

To investigate in vitro drug release from the prepared inserts, a certain amount of each formulation and a commercial CIP eye drop containing an equal volume of CIP were placed in a donor compartment containing 500 μL of PBS (pH 7.4) and separated by a dialysis membrane from the receptor medium containing 49.5 mL of PBS. The system was kept at 37 °C and stirred at 100 rpm. Samples were removed from the receptor medium at regular intervals and the whole medium was replaced with fresh PBS. Quantification of CIP in the receptor phase was performed using HPLC.

### 2.9. Release Mechanism

To predict the mechanism responsible for drug release, in vitro release data were fitted into kinetic models of zero-order, first-order, Higuchi, and Korsmeyer-Peppas, and the model with the highest correlation was used to describe the main release mechanism.

### 2.10. In Vitro Cytotoxicity

The L929 cell line (mouse fibroblasts) was placed in a sterile plate containing complete medium consisting of DMEM/F12 medium (1:1 *v*/*v*, Gibco, Paisley, Strathclyde, UK) supplemented with 10% fetal bovine serum (Gibco Invitrogen Srl, Milan, Italy), 100 U/mL penicillin, and 100 μg/mL streptomycin and cultured in a humidified incubator set at 37 °C and containing 5% CO_2_ for 24 h.

The cultured cells were implanted in 96-well plates. A row of the plate that did not contain any formulation was considered the control. Samples with different concentrations of CIP were added to the other rows. The culture medium was removed and the plates washed with PBS. Trypsin was added to free attached cells, and 10 μL of MTT solution (5 mg/mL) was then added to the wells. Control samples were prepared by adding DMSO to the cultured cells to completely inhibit the cells. The plates were incubated for 4 h under standard conditions and the absorbance of the samples was measured at 570 nm using a UV spectrophotometer. The percentage cell viability was calculated using Equation (3).

Cell viability (%) = OD sample/OD control × 100 (3)

### 2.11. Irritancy Test and In Vivo Study in Rabbits

New Zealand male rabbits weighing 3.5–4 kg were used for irritancy testing. A piece of the prepared CIP-loaded insert and 100 µL of sterile PBS were administrated to the ocular sac of the rabbits’ left eyes; the rabbits’ right eyes received the same volume of PBS as a control. Irritation was examined four days after administration of the inserts and scored using the scoring system defined in ISO-10993-10, which is classified based on clinical observations of redness, tearing, and discomfort in the eyes (Table 1).

In vivo studies were performed on healthy rabbit eyes. First, the eyes were washed with sterile PBS and then 20 mg of CIP-OG and CIP-O nanofibrous inserts, along with PBS and one drop of commercial CIP 0.3% eye drop, were placed inside the rabbit’s eye sac. Tear fluid was sampled at specific intervals. HPLC was used to quantify the drug, and the concentration of the drug in the tear film was calculated using regression equations. The procedure was conducted in accordance with the resolution on the animal’s study and approved by the Ethics Committee of Kermanshah University of Medical Sciences (KUMS.REC.1395.399).

### 2.12. Statistical Analysis

Kruskal-Wallis and T-tests were used for statistical data analysis. The significance level was considered to be 0.95 and the difference was presumed significant at *p* < 0.05.

## 3. Results

### 3.1. Preparation of Ocular Inserts

**Figure 1:**
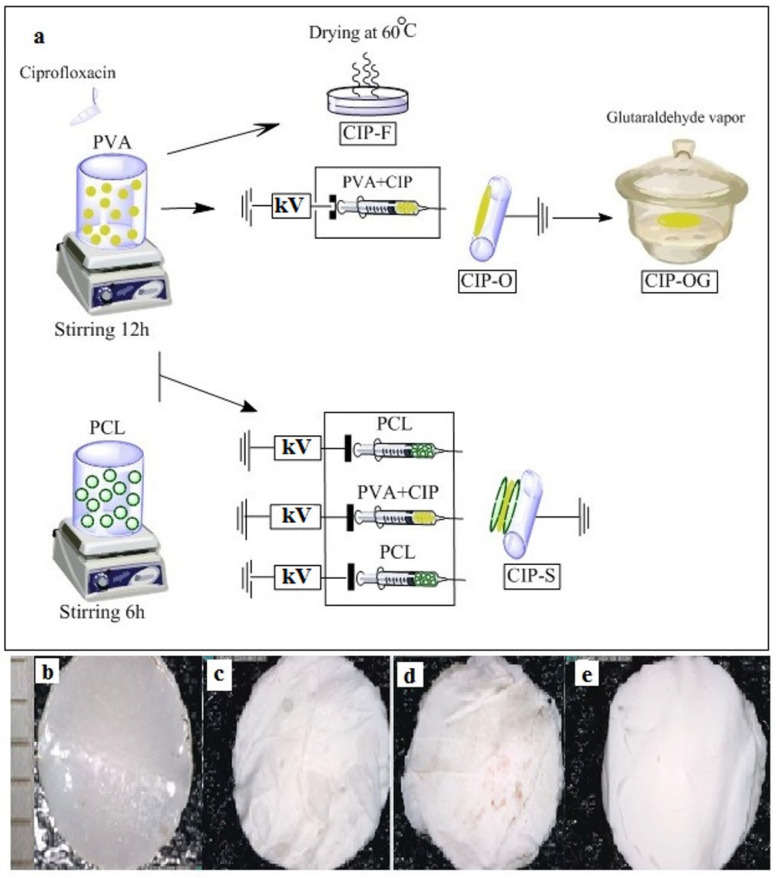
Schematic representation of the preparation process (**a**) of ocular inserts, including CIP-F (film insert), CIP-O (one-layered nanofiber), CIP-OG (GA crosslinked one-layered nanofiber), and CIP-S (multi-layered nanofiber with sandwich structure). Macrograph of CIP-F (**b**) CIP-O (**c**), CIP-OG (**d**), and CIP-S (**e**) formulations captured using the Dino-Lite Digital Microscope (Dino-Lite Premier, Taiwan). Scale: 1 mm.

Figure 1a represents the flow diagram of the process of nanofibrous formulation preparation. CIP-loaded nanofibrous inserts (CIP-O, CIP-OG, and CIP-S) were prepared by electrospinning PVA–CIP solutions. The cross-linked CIP-OG formulation was prepared by exposing CIP-O to GA vapor for 10 h. GA cross-linking led to a slight change in the color of the insert and a reduction in its dimensions. This discoloration and shrinkage of CIP-OG may be due to the linkage of free hydroxyl groups of PVA to GA [16]. As shown in Figure 1b, the film-structured insert prepared using the solvent casting method had a smooth surface, uniform texture, and off-white color. CIP-O and CIP-OG nanofibrous inserts are shown in Figure 1c,d. PCL was used to fabricate the outer layers covering a middle CIP-loaded PVA layer in the CIP-S formulation that also had a suitable visual appearance, as shown in Figure 1e.

### 3.2. Characterization of Ocular Inserts

#### 3.2.1. Weight, Thickness, and Drug Content Uniformity

Table 2 represents the mean weight, thickness, and drug content efficiency. The film and nanofibrous ocular inserts had uniform weights, with the mean ranging between 19.50 and 22.77 mg for 1 × 1 cm^2^ pieces. The thickness of the CIP-F insert was greater than that of the CIP-loaded nanofibers. CIP-F was the thickest (0.210 ± 0.009 mm), while CIP-O was the thinnest (0.106 ± 0.002 mm).

#### 3.2.2. Folding Endurance and Tensile Strength Testing

Folding endurance is an indication of the flexibility of the inserts, which is an influential factor during the preparation and administration of ocular inserts. Based on the data presented in Table 2, the folding endurance of different formulations ranged between 203 and 224. Generally, nanofibrous inserts had higher flexibility compared with the film formulation. Of note among the nanofibers, the multilayer insert (CIP-S) had the highest folding endurance. The results of the tensile testing showed that the CIP-F formulation broke faster than the other formulations, with a break time of 4.06 min and an elongation of 27% at the break. The CIP-S formulation, with a break time of 12.20 min and an elongation of 71.69% at the break, had the highest resistance against breaking (Table 2).

#### 3.2.3. Swelling, Moisture Loss, and Uptake Tests

Based on the values measured at the different time intervals presented in Table 3, the CIP-F film and the CIP-S sandwich-structured nanofiber had the lowest and highest degrees of swelling, respectively. Table 3 indicates the moisture loss and uptake percentages of different formulations. Moisture loss was found to vary between 0.68 and 1.08% when moisture uptake was between 0.72 and 1.28%. Among the prepared formulations, CIP-S had the lowest percentage of moisture loss and absorption compared with the other formulations, which could be due to lower penetration of water through the hydrophobic PCL coating.

### 3.3. Antibacterial Efficacy of Ocular Inserts

**Figure 2:**
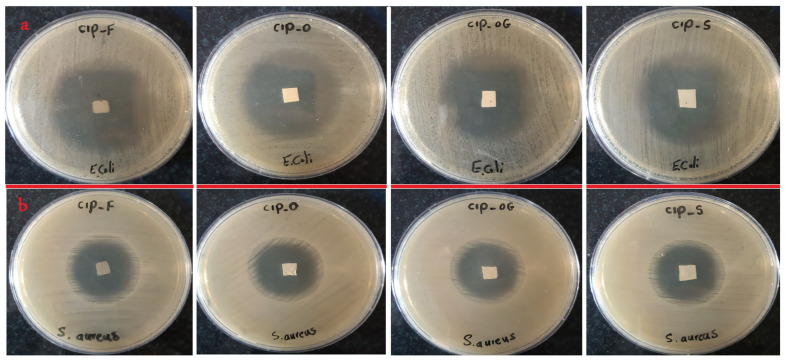
Inhibited growth zones and antibacterial efficacy of ocular inserts containing 1000 µg of the drug with different formulations against *E. coli* (**a**) and *S. aureus* (**b**).

The antibacterial efficacy of each prepared insert against both Gram-positive *S. aureus* and Gram-negative *E. coli* bacteria was examined by measuring growth inhibition zones surrounding the formulations (Table 4). It can be concluded that the antibacterial activities of the CIP-loaded film and the nanofibrous inserts with different formulations were relatively higher against *E. coli* than *S. aureus*; however, all inserts were efficacious at inhibiting both species’ growth. Figure 2 displays the results of in vitro antibacterial efficacy tests.

### 3.4. FTIR Spectroscopy

**Figure 3:**
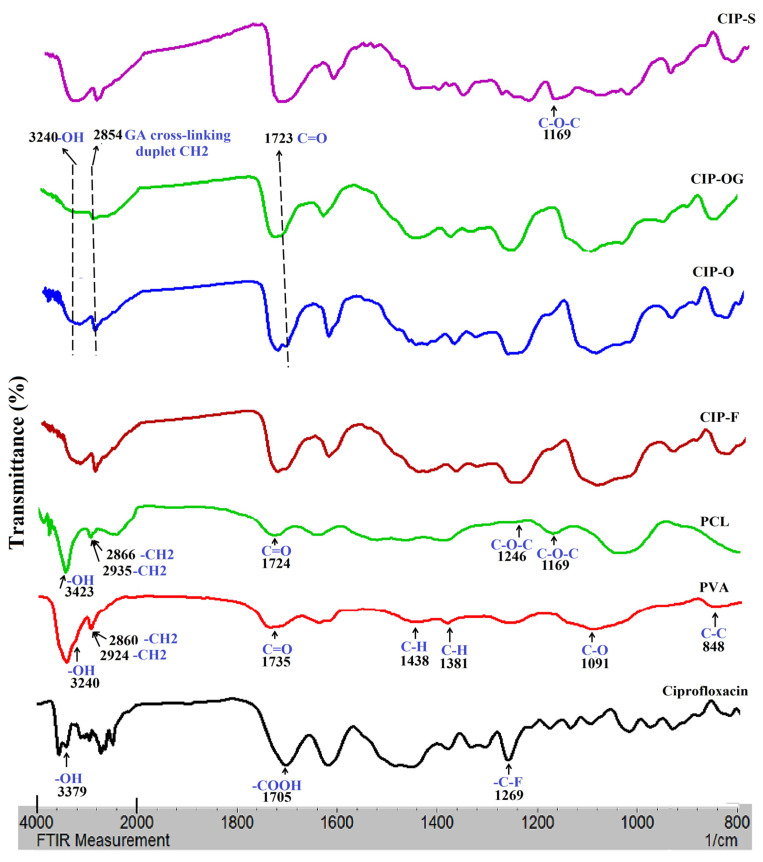
FTIR spectra of ciprofloxacin, PVA, PCL, PVA film insert (CIP-F), single-layered PVA nanofiber (CIP-O), GA-crosslinked PVA nanofiber (CIP-OG), and multi-layered PCL-coated PVA nanofiber with sandwich structure (CIP-S).

Figure 3 represents the FTIR spectra for pure CIP, PVA, PCL, CIP-F, CIP-O, CIP-OG, and CIP-S. Examining the spectra of the film and nanofibrous inserts shows a peak at approximately 3379 cm^−1^, which corresponds to the OH groups in PVA and CIP. The peaks that appear at 1269 cm^−1^ are attributed to the C-F groups of CIP, and appear in the spectra of all inserts. Characteristic peaks of PVA are also observable, with a slight shift in the spectra of all ocular inserts, including an extended characteristic peak at around 3400 cm^−1^, which corresponds to OH groups. Peaks are also observed at 2927 and 2875 cm^−1^, associated with asymmetrical and symmetrical C-H bond stretching. A peak with a slight shift in the spectra of all inserts is also observed at approximately 848 cm^−1^, indicating the presence of C-C bonds in PVA.

The intensity of the OH peak at 3240 cm^−1^ is decreased in the spectrum of the CIP-OG nanofiber. This may be due to the interaction of PVA hydroxyl groups with GA and the formation of acetal rings and ether bonds during the crosslinking process [17]. In addition, changes related to the stretching vibrations of CH2 groups in cross-linked CIP-OG nanofibers were observed at 2854 cm^−1^. A shift in the C-O-C stretching peak toward higher frequencies, which occurred due to the formation of C-O-C bonds as a result of acetal and ether bonding during the reaction between the hydroxyl groups of PVA and GA [18], was detected. The CIP-S spectrum had a peak at 1169 cm^−1^ corresponding to the symmetric stretching of C-O-C groups in PCL.

### 3.5. Morphology of Ocular Inserts

**Figure 4:**
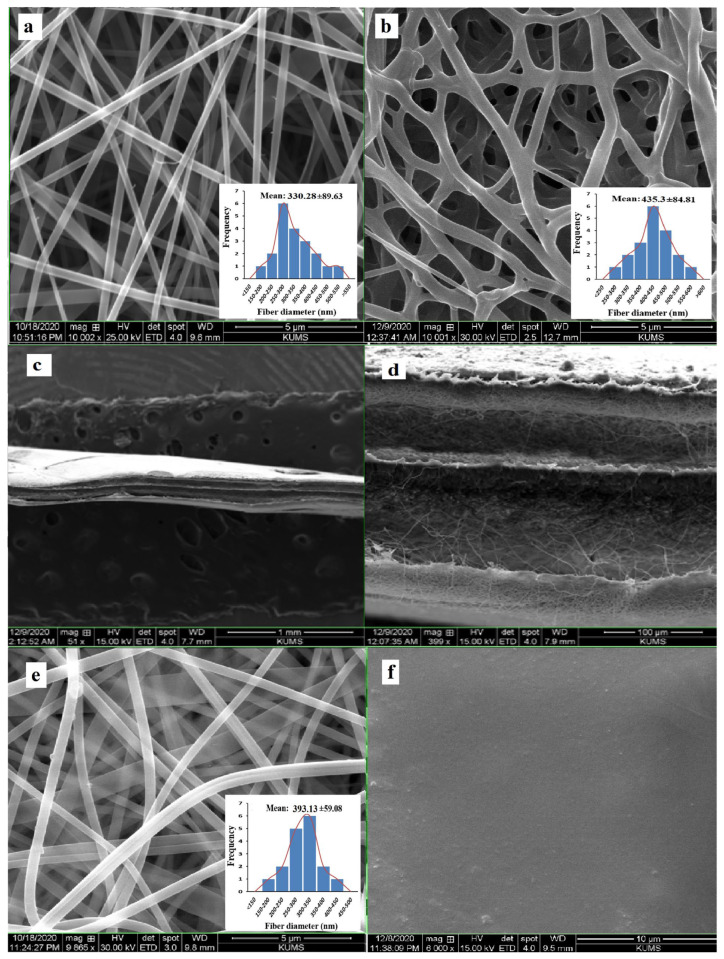
SEM images of single-layered non-crosslinked PVA nanofiber (CIP-O) at ×10,000 magnification (**a**), single-layered GA-crosslinked nanofiber (CIP-OG) at ×10,000 magnification (**b**), a cross-section of sandwich-structured nanofiber (CIP-S) at ×50 (**c**) and ×400 (**d**) magnifications, drug-free PCL layer of CIP-S nanofibers at ×10,000 magnification (**e**), and CIP-F film insert at ×6000 magnification (**f**).

The morphologies of all formulations and the diameters of fibers in nanofibrous inserts were determined using SEM. Nanofibers were aligned randomly in the CIP-O and had a mean diameter of 330 ± 89 nm (Figure 4a). The average diameter of GA cross-linked nanofiber (CIP-OG) was 435 ± 84 nm, which was significantly higher than the diameter of non-crosslinked CIP-O (Figure 4b). Figure 4c,d indicate the cross-sectional SEM images of CIP-S sandwich-structured nanofibers at different magnifications. The outer layers consist of PCL nanofibers and the middle layer comprise the CIP-loaded PVA nanofiber. It is obvious that the outermost PCL layers were beadless, with an average diameter of 393 ± 59 nm, which was higher than the mean diameter of the drug containing the PVA layer in the middle (Figure 4e). Figure 4f shows the SEM image of the film formulation (CIP-F), which had a smooth and non-cracked surface with no fractures or cavities.

### 3.6. In Vitro Release Study

**Figure 5:**
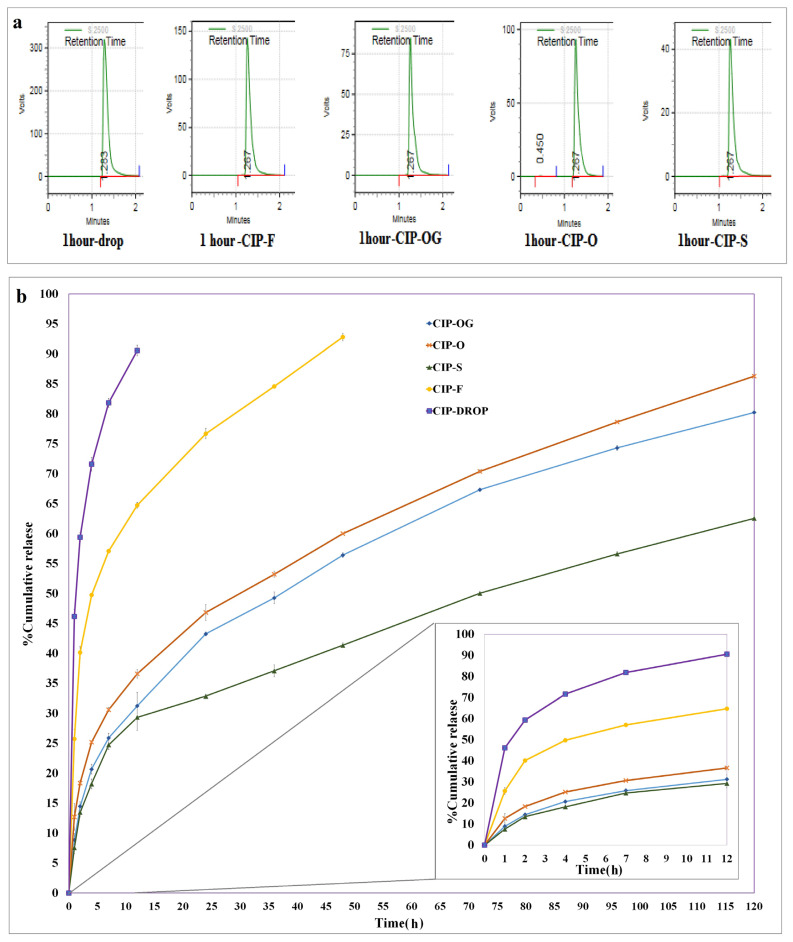
HPLC peaks (**a**) of different formulations obtained 1 h after administration during in vitro studies. Comparisons of in vitro release (**b**) of CIP from CIP-F, CIP-O, CIP-OG, CIP-S, and CIP commercial eye drop (CIP-Drop) in PBS (pH = 7.4) at 37 °C.

Quantification was performed using HPLC. Figure 5a showed the chromatographic peaks of the different formulations. Figure 5b shows the cumulative percentages of the drugs released from the commercial eye drop and the prepared ocular inserts during the in vitro study.

The eye drop formulation released 59.40 ± 0.70% of its drug content in the first 2 h, while the CIP-F film-casted insert released 64.74 ± 0.49% of the drug during the first 12 h. A similar study reported that ocular inserts of ethyl cellulose and gelatin prepared using the solvent casting method provided a steady release of CIP for up to 12 h [19]. Another study reported a 280–440 min release of CIP from ocular film inserts of methylcellulose, hydroxypropyl methylcellulose, hydroxypropyl cellulose, and Eudragit polymers [20].

Among the nanofiber formulations, CIP-O had the most rapid release profile, while CIP-S had the slowest release profile, releasing 29.35 ± 0.25% of the drug over 12 h.

CIP-OG had a relatively slower release rate, releasing 31.25 ± 2.19% of its drug content over 12 h compared with CIP-O, which released 36.59 ± 0.71% of the drug over the same interval.

The total drug released from the eye drop formulation and CIP-F reached a maximum of almost 90.57 ± 0.89% within 12 h and 92.82% within 48 h. Nanofibrous inserts released drugs for 120 h, with a cumulative percentage of 62.55 ± 0.25%, 80.24 ± 0.15%, and 86.98 ± 0.20%, respectively, for CIP-S, CIP-OG, and CIP-O formulations. In another similar study, cellulose acetate was blended with PVA for the fabrication of electrospun nanofibrous ocular inserts of itraconazole, and was reported to release the drug over a prolonged period of 55 days [21]. A PVA-based ocular insert of gatifloxacin was developed in another study, which showed controlled release of the drug over 24 h [22].

### 3.7. Release Mechanism

Fitting the release data into different models indicated that CIP-F followed the first-order kinetic model, with a correlation coefficient (R2) of 0.9621. The release was mainly governed by matrix degradation. If a homogeneous matrix erosion process is assumed and the release behavior follows the law of first order reactions, the expression of the polymer molecular weight function of time can be obtained [23]. CIP-O, CIP-OG, and CIP-S followed Higuchi-like release behavior, with R2 of 0.9834, 0.9888, and 0.9206. The diffusion phenomenon is the dominant mechanism behind the release of drugs from the formulations following the Higuchi model.

### 3.8. In Vitro Cytotoxicity

**Figure 6:**
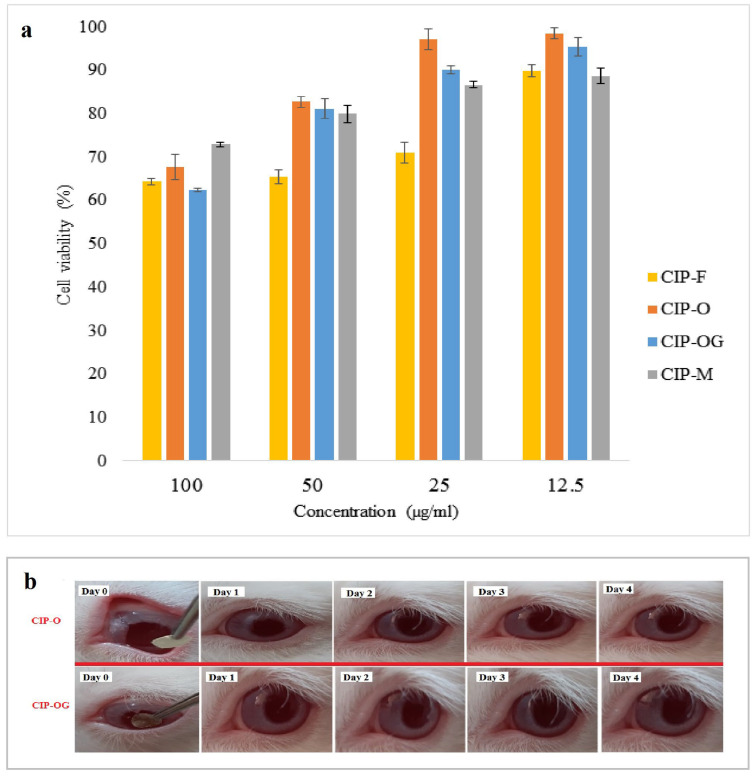
(**a**) Cell viability of different ocular inserts containing different concentrations of CIP measured using the MTT method. Data are expressed as mean ± SD of three separate experiments (*n* = 3). (**b**) The appearance of rabbits’ eyes treated with CIP-O and CIP-OG ocular inserts over 4 days of irritancy testing.

Cell viability was determined using the MTT assay. Ocular safety evaluation mainly included cytotoxicity test [24]. According to ISO-10993-5, cell viability above 80% is considered non-toxic, between 80 and 60% is weakly toxic, 60–40% is moderately toxic, and below 40% is highly toxic [25]. Figure 6a shows cell viability following administration of the inserts, based on drug concentrations. A formulation with a low level of cytotoxicity (cell viability >70%) is considered safe for ocular drug delivery. The highest percentage of cell viability for each formulation was associated with the lowest concentration of the drug (12.5 µg/mL). The CIP-O formulation had the highest cell viability at all concentrations. There were no significant differences between cell viability of cross-linked CIP-OG and non-crosslinked CIP-O nanofibers; therefore, both CIP-O and CIP-OG ocular inserts were subjected to animal studies.

### 3.9. Irritancy Test and In Vivo Study in Rabbits

CIP-O and CIP-OG nanofibers were subjected to animal studies due to their appropriate physicochemical properties that made them convenient for placement in rabbits’ eyes. CIP-S was ruled out for the animal study because of its higher dimensions compared with single-layered formulations—arising from coating with PCL layers—along with lower mucoadhesive properties that could lead to faster elimination of the formulation from the eye.

In the present work, inserts were placed in the conjunctival sac of rabbits’ eyes without the requirement for any surgery, anesthesia, or sutures. Irritation scores for the irritancy test remained at 0 over four days of evaluation. Figure 6b shows that there was no detectable redness or turbidity in the cornea, the iris and conjunctival blood vessels looked normal, and no swelling was observed in the eyelids.

**Figure 7:**
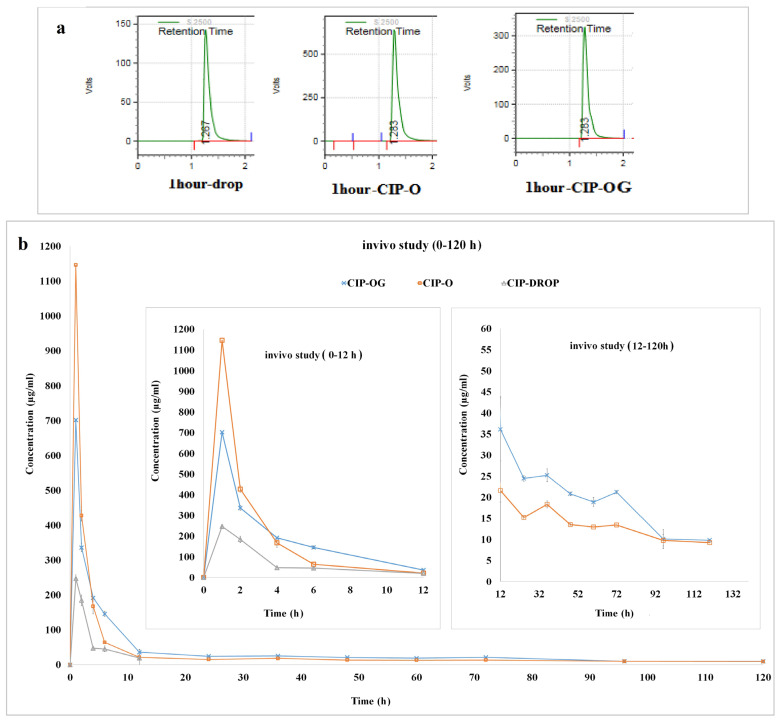
(**a**) HPLC peaks obtained 1 h after administering the different formulations during in vivo study. (**b**) In vivo release of CIP from CIP-O and CIP-OG ocular inserts and CIP 0.3% *w*/*v* eye drops and tear concentrations in rabbits’ eyes over 120 h.

Figure 7a showed the chromatographic peaks of CIP during evaluation, and cumulative percentages of the released drug in tear film are presented in Figure 7b. The drug concentration achieved using the eye drop formulation in tear fluid reached a maximum of 247.7 ± 10.4 μg/mL 1 h after administration and dropped to 19.07 ± 3.1 μg/mL after 12 h. Of note, the concentration of drug released from the eye drop dropped below the limit of detection—as determined by HPLC—after 12 h.

The pharmacokinetics of drug release from CIP-loaded ocular inserts and the CIP eye drop are provided in Table 5. All formulations reached the maximum concentration (C_max_) of released CIP in tear fluid 1 h after administration. The C_max_ of the drug released from CIP-OG and CIP-O formulations were 702.31 ± 12.13 μg/mL and 1146.11 ± 32.35 μg/mL, respectively, and were followed by a gradual and steady release of the drug into the lacrimal fluid for 120 h. AUC_0–120_ were 859.11 ± 16.60, 3884.098 ± 15.97, and 4314.99 ± 1.41 for the CIP eye drop, CIP-O, and CIP-OG formulations, respectively. MRT of CIP eye drop, CIP-O, and CIP-OG formulations were 3.30 ± 0.02 h, 23.30 ± 0.50 h, and 27.61 ± 0.97 h, respectively.

## 4. Discussion

We succeeded in fabricating high-strength flexible ocular inserts of CIP as biodegradable, biocompatible, and anti-infective formulations for the treatment of ocular infections. The film and nanofibrous ocular inserts displayed weight uniformity. The standard deviations for weight and thickness confirmed the uniformity of the formulations and each formulation had a thickness of less than 0.220 mm.

All formulations showed more than 96% loading efficiency, with uniform loading across the fabricated mats. High drug content efficiency has the advantage of increasing loaded drug content in an ocular insert with reduced size, which will eventually enhance patient satisfaction because of the ease of administration, and reduced inflammation and irritation caused by the formulation.

All inserts showed acceptable folding endurance; therefore, they are considered to have appropriate flexibility to be placed in the ocular sac. The optimum range for folding endurance is considered to be between 200 and 300 [26]. Values above 300 may cause excessive insert stiffness, while values below 200 could be an indicator of insufficient strength of inserts to maintain their integrity against eye movement and blinking. As expected, the CIP-S nanofiber formulation with the flexible PCL-based outer layers had the highest tensile strength. The lower strength of CIP-F could be attributed to the preparation process. On the other hand, the GA-crosslinked CIP-OG nanofiber had a higher tensile strength compared with the non-crosslinked CIP-O because of the development of a rigid bonding network between fibers at intersections which could enhance the mechanical properties of crosslinked nanofibers [8,27]. Determining the degree of swelling is crucial to understanding the drug release behavior of inserts. The higher swelling caused by the film formulation could be due to higher water absorption by film-casted inserts compared with inserts with nanofibrous structures. Among the electrospun nanofibrous inserts, the CIP-S sandwich-structured nanofiber had the lowest degree of swelling, owing to the presence of relatively hydrophobic PCL layers in its structure giving it a lower ability for water absorption compared with PVA [5].

To evaluate the physical stability and uniformity of ocular inserts in dry and humid environments, moisture loss and uptake values were measured. Less than 2% change in weight suggests that the developed ocular inserts have acceptable stability under different conditions.

CIP is a second-generation fluoroquinolone antibiotic with a wide range of activities against Gram-positive and Gram-negative bacteria. The mechanism of action underlying the antibacterial effect of this drug involves the inhibition of DNA-gyrase, a critical enzyme involved in the proliferation of bacterial chromosomes. Similar to other fluoroquinolones, CIP contains a piperazine group in the 7th position of the 4-quinolone nucleus, which results in antibacterial activity against the Gram-positive and Gram-negative bacteria responsible for ocular infections [28]. The results of the antibacterial efficacy of ocular inserts showed that the prepared inserts can effectively prevent the growth and proliferation of bacteria in an infected eye.

The appearance of drug-specific FTIR peaks in the spectra of formulations confirms the presence of the drug in the structure of films and nanofiber formulations. Some of the characteristic CIP peaks were had a slight and negligible shift in the spectra of all inserts.

SEM analysis of CIP-loaded nanofibrous inserts showed uniform structures with no bead defects. The average diameter of the GA cross-linked nanofiber (CIP-OG) was significantly higher than the diameter of the non-crosslinked CIP-O. The increased diameter seems to be due to the swelling of the nanofibers during the crosslinking process. The effect of exposure to GA vapor on the diameter of PVA/chitosan nanofibers was previously investigated [17]. The study found that the diameter of the nanofibers increased during crosslinking, in accordance with the results obtained in our study. Generally, multi-layered nanofibers containing beadless PCL and PVA layers had higher average diameters compared with single-layered nanofibers.

Multiple studies have investigated the in vitro release of CIP from ocular preparations [19,20]. Based on the data reported in these previous studies, the film and nanofiber formulations were expected to exhibit a two-phase release behavior: a burst phase followed by controlled drug release. PVA-based nanofibers were used for the delivery of CIP in multiple previous studies, which reported sustained and prolonged drug release [29,30]. The rate of drug release from CIP-loaded nanofibrous inserts was significantly lower than that from film formulations. Comparable results were obtained in a study that developed ocular preparations of dexamethasone using solvent casting and electrospinning methods. This study reported that PLA/PVA electrospun nanofibers performed better than film-casted inserts by eluting the drug more gradually over 36 h [26]. Among the nanofiber formulations, CIP-O had the most rapid release profile, while CIP-S had the slowest. In fact, the presence of hydrophobic nanofibrous coating layers of PCL around the PVA hydrophilic layer with a sandwich structure in this formulation could control the release rate and thus resist the rapid release of the drug from the insert. Sandwich-structured nanofibers have been used in multiple studies to prolong drug delivery [31,32]. Controlled release of vancomycin and gentamicin antibiotics over 24 days was achieved using sandwich-structured nanofibers [31]. We previously observed the same results, where sandwich-structured nanofibers showed slower ofloxacin release, without a burst phase, compared with one-layered nanofibers [32]. CIP-OG had a relatively slower release rate compared with CIP-O over the same period. This may be due to the formation of crosslinking bonds between PVA hydroxyl groups by GA, which reduced the rate of swelling as well as drug release [26]. We obtained similar results in our previous study, where GA-crosslinking led to formation of a network-chain structure with a lower degree of swelling and decreased the amount of released azithromycin and eliminated the burst phase. Therefore, the release of azithromycin was slower in cases with crosslinked fibers compared with non-crosslinked formulations [8].

Fitting the release data into different models indicated that CIP-F followed the first-order kinetic model. This result was in accordance with that of a previous study, which reported first-order kinetics for film formulations [33]. CIP-O, CIP-OG, and CIP-S followed a Higuchi-like release behavior. Most of the controlled-release polymeric matrices followed the same kinetics. In fact, the release mechanism included two phases: the drug loaded onto the surface of fibers initially diffused into the receptor medium and then the medium penetrated the porous structure of the nanofibers and dissolved and gradually released the drug.

The results of in vitro cytotoxicity tests showed that each of the prepared inserts had acceptable biocompatibility for ocular application. Increasing the concentration of CIP loaded in the formulations reduced cell viability.

CIP-O and CIP-OG nanofibers were subjected to animal studies. The results of the irritancy test conducted prior to the in vivo study showed that according to the ISO 10993-10: 2010 scoring system, no sign of irritancy or discomfort such as blinking or severe tearing was observed in rabbits’ eyes following the administration of CIP-O and CIP-OG formulations. CIP-O and CIP-OG nanofibers provided long-term release of the drug in the eyes without causing any harm to the eyes. The results were in accordance with a previous study that developed ocular film inserts of CIP and sodium alginate using polyvinyl acetate and eudragit polymers [34]. The results of in vivo evaluation in the mentioned study indicated that the drug concentration in rabbit lacrimal fluid reached the minimum inhibitory concentration (MIC) of CIP immediately and remained at this level for 4–4.5 days. Both formulations had increased C_max_ compared with eye drops, but the slightly higher drug concentration in the case of CIP-O can be explained by its higher degree of swelling compared with CIP-OG nanofiber. The low C_max_ value of the eye drop can be attributed to its rapid elimination from the surface of the eye because of tears turnover. The AUC_0–120_ values for CIP-O and CIP-OG formulations were approximately 4.5–5.0 times higher than those of eye drops. MRT of CIP-O and CIP-OG formulations were about 7.0–8.3 times higher than that of the eye drop. Generally, nanofibrous inserts had the highest AUC_0-120_ and MRT, which can be explained by the encapsulation of the drug in the porous structure leading to stable and long-term release of the drug from the prepared nanostructures. MIC90 is defined as the minimum concentration that inhibits the growth of 90% of common Gram-positive and Gram-negative bacteria that cause bacterial infections. MIC90 of CIP is approximately 0.5 µg/mL [35]. Results of the in vivo studies suggested that the commercial CIP eye drop formulation could maintain the drug concentration in lacrimal fluid at levels higher than the MIC90 for only 12 h, while ocular inserts can maintain the concentration in the lacrimal fluid at levels higher than the MIC90 for up to 120 h. In a similar study, an in situ gelling system of pefloxacin mesylate was used to achieve long-term drug release at concentrations higher than the MIC90 concentration in rabbit’s tears for 12 h [36].

## 5. Conclusions

Due to the recent significant increase in the prevalence of ocular bacterial infections that can lead to consequences such as irreversible blindness, along with the inability of conventional ocular delivery systems to achieve desired therapeutic drug concentrations in the eye, this study aimed to design controlled-release film-structures and nanofibrous inserts to improve the topical ocular drug delivery of the antibacterial drug ciprofloxacin. Ciprofloxacin-loaded ocular inserts possess suitable physicochemical properties for ocular application, including uniformity, flexibility, strength, stability under dry and humid conditions, and similar acidity to tears. The formulations showed in vitro antibacterial efficacy against *S. aureus* and *E. coli*, and showed no significant cytotoxicity during MTT assays using L929 fibroblast cells. The results of in vitro studies indicated long-term and slow release of ciprofloxacin from the film and nanofibrous formulations for 48 h and 5 days, respectively, while the commercial eye drop provided effective concentrations of the drug in the lacrimal fluid for up to 12 h. Compared with the commercial eye drop, higher intraocular bioavailability and increased residence time, long-term and targeted release of the drug in the eye can be provided using nanofibrous ocular inserts. In conclusion, ocular inserts are suitable alternatives for conventional eye drops, reducing the frequency of administration and enhancing patient compliance.

## Figures and Tables

**Table 1:** Grading eye irritation in rabbits.

Score	Clinical Observations
**0**	No sign of redness or cornea turbidity; conjunctival blood vessels look normal; no swelling; normal blinking; no tearing
**1**	The cornea has scattered areas of turbidity; some conjunctival blood vessels look hyperemic; any higher-than-normal swelling; slight tremors; any abnormal tearing
**2**	Details of the iris are slightly blurred; ocular hemorrhage; ocular aberration; iris shows no reaction to light; swelling, with slight dilation of the eyelid
**3**	Glossy areas are detectable; iris details are not recognizable; pupil is difficult to detect; scattered dark red spots are detectable; swelling, with the eyelid half-closed
**4**	Opaque cornea; iris is not recognizable due to high turbidity; swelling, with the eyelid almost closed

**Table 2:** Physicochemical properties of the prepared ocular inserts (mean ± SD).

Formulation	Weight Uniformity (mg)	Thickness (mm)	Folding Endurance (Times)	Drug Content Efficiency (%)	Elongation at Break (%)	Time to Break (Min)	Tensile Strength (MPa)
**CIP-F**	22.77 ± 0.20	0.210 ± 0.009	203 ± 5	98.1 ± 0.7	27.01	4.06	1.15 ± 0.11
**CIP-O**	19.50 ± 0.12	0.106 ± 0.002	213 ± 3	96.2 ± 1.5	29.70	6.25	1.96 ± 0.03
**CIP-OG**	20.15 ± 0.18	0.108 ± 0.004	210 ± 1	97.9 ± 1.6	48.12	10.13	2.43 ± 0.02
**CIP-S**	21.77 ± 0.25	0.178 ± 0.007	224 ± 8	98.4 ± 0.8	71.69	12.20	2.62 ± 0.13

**Table 3:** Degree of swelling, moisture loss, and uptake of the prepared ocular inserts (mean ± SD).

Formulation	Moisture Loss (%)	Moisture Uptake (%)	Swelling (%) 3 h	Swelling (%) 6 h	Swelling (%) 12 h
**CIP-F**	1.08 ± 0.05	1.28 ± 0.11	190.2 ± 2.5	207.6 ± 3.1	232.2 ± 2.8
**CIP-O**	0.93 ± 0.01	1.04 ± 0.03	139.1 ± 2.5	154.6 ± 3.3	172.2 ± 3.7
**CIP-OG**	0.82 ± 0.03	0.94 ± 0.01	129.1 ± 1.6	143.9 ± 3.4	161.3 ± 2.2
**CIP-S**	0.68 ± 0.01	0.72 ± 0.01	117.6 ± 2.4	133.4 ± 4.3	137.1 ± 6.6

**Table 4:** Diameters of inhibited growth zones surrounding different ocular inserts against *E. coli* and *S. aureus* obtained using the antibacterial efficacy test.

Formulation	Diameter of Inhibited Growth Zone (mm) Against	
	*E. coli*	*S. aureus*
**CIP-F**	4.8 ± 0.1	3.3 ± 0.1
**CIP-O**	4.7 ± 0.1	3.5 ± 0.1
**CIP-OG**	4.5 ± 0.2	3.7 ± 0.2
**CIP-S**	4.8 ± 0.1	3.5 ± 0.1

**Table 5:** Pharmacokinetics of CIP-O and CIP-OG ocular inserts and CIP 0.3% *w*/*v* eye drop in rabbit eyes.

Formulation	C_max_ (μg/mL)	AUC_0-120_	MRT (h)
**CIP-OG**	702.31 ± 12.13	4314.99 ± 1.41	27.61 ± 0.97
**CIP-O**	1146.11 ± 32.35	3884.09 ± 15.97	23.30 ± 0.50
**CIP-DROP**	247.70 ± 10.40	859.11 ± 16.60	3.30 ± 0.02

## Data Availability

Not applicable.
